# Activity Workstations in High Schools: Decreasing Sedentary Behavior Without Negatively Impacting Schoolwork

**DOI:** 10.3389/fpsyg.2022.929564

**Published:** 2022-06-24

**Authors:** June J. Pilcher, Timothy L. Hulett, Paige S. Harrill, Jessie M. Cashman, G. Lawson Hamilton, Eva Diaz

**Affiliations:** Department of Psychology, Clemson University, Clemson, SC, United States

**Keywords:** sedentary behavior, physical activity, high school, READ 180 program, academic performance, school-related subjective measures

## Abstract

High school students are at risk for increased sedentary behavior due in part to a decrease in physical activity throughout adolescence and to required sedentary behavior during much of the school day. The purpose of the current study is to examine the impact of using activity workstations in a high school English class for struggling readers. Twenty high school students participated in the study. The participants completed a 16-week study where each participant used an activity workstation for 8 weeks and a traditional desk for 8 weeks in a crossover design for a 40-min period during normal class. They responded to a series of subjective questions about reading and schoolwork at the beginning and end of each 8-week session and followed the READ 180 program designed to help struggling readers during the study. The results indicated that academic performance increased in both desk conditions during the study and from the beginning to the end of the study. In addition, there was a significant improvement in items in the subjective survey related to reading, motivation, and schoolwork in both desk conditions across the study. The current results suggest that using an activity workstation in the classroom did not negatively affect academic performance or students’ perceptions of working on academic assignments compared to the traditional desk condition. These results indicate that activity workstations could be implemented in classrooms to provide students with a non-sedentary option during the school day thus increasing physical activity in students.

## Introduction

Many school-age children do not meet the recommended 60 min of physical activity each day (World Health Organization, 2016). Furthermore, the number of children meeting the recommendations for daily activity decreases throughout adolescence (Cornelius et al., 2020). As such, adolescents are at particular risk of increased sedentary behavior which increases their likelihood of becoming overweight and obese (Ogden et al., 2012) as well as increasing their long-term health risk (Kohl and Cook, 2013). Given that adolescents spend many hours each day during the school year in a classroom setting, it is important to consider how the classroom environment could contribute to sedentary behavior as well as opportunities for physical activity. The traditional desk in most classroom settings requires sedentary behavior from students. One way to reduce sedentary behavior in the classroom and to increase light physical activity is through incorporating activity workstations allowing students to engage in classroom activities or to complete desk-based tasks while being physically active.

Some studies have investigated the potential impact of activity workstations in a classroom setting on physical activity and classroom behavior in students. Activity workstations increase low-intensity physical activity (Fedewa et al., 2017) and energy expenditure (Torbeyns et al., 2017) when compared to traditional classroom seating. Similar results were found when placing standing desks in a classroom setting (Pickens et al., 2016), suggesting that students could benefit from the opportunity to use a classroom desk arrangement that encourages some type of physical activity other than simply being seated. Fedewa et al. (2017, 2018) also found that on-task behavior was not negatively affected by activity workstations in high school English classes and high school special education classes. In addition, in a meta-analysis examining the impact of standing desks in a classroom on student behavior such as concentration and inattention, there were no significant changes due to the use of standing desks (Minges et al., 2016).

Although a number of studies have suggested that moderate-to-vigorous physical activity during the school day can improve academic performance (Fedewa and Ahn, 2011; Donnelly et al., 2016; Alvarez-Bueno et al., 2017), there is less information on the potential impact of light physical activity on academic achievement. One study with young adolescents using bike desks in a classroom found no significant change in academic performance (Torbeyns et al., 2017). Similarly, a systematic review of dynamic seating interventions in classrooms found that none of the interventions had a detrimental effect on academic outcomes (Rollo et al., 2019), whereas a meta-analysis examining the impact of a variety of types of physical activity interventions in the classroom found a moderate improvement in language skills but no change in mathematics or grade point average (Haverkamp et al., 2020).

It is also important to consider the potential impact of using activity workstations in the classroom on meta-cognitive variables. For example, if using workstations results in some type of positive outcome or feeling, this could encourage individuals to engage in physical activity in the future and across the lifespan (Pilcher and Baker, 2016). Unfortunately, little research has examined the potential effect of using activity workstations on meta-cognitive variables (Rhodes et al., 2012). One study found that using activity workstations in a work setting positively impacts stress and affect (Sliter and Yuan, 2015). In other studies, when college students used workstations while completing laboratory-based tasks, there was an improvement in positive affect, motivation, and morale (Pilcher and Baker, 2016) as well as a decrease in sympathetic reactivity during stressful tasks (Pilcher et al., 2022). In addition, it is important to note that performance on the laboratory-based tasks in these studies did not suffer due to using the activity workstations.

The potential impact of using activity workstations in classes designed for high school students who are struggling to meet the academic demands of their grade level has not yet been investigated. One group of particular concern is students who are struggling readers since the ability to read and understand technical documents is essential for success in many workplaces (Friedman, 2006). Research suggests that 90–95% of struggling readers can improve their reading skills if they receive appropriate interventions (Drummond, 2005). One type of reading intervention is Scholastic’s READ 180 program which can be used in students reading below expected proficiency levels in grades 4 through 12. The READ 180 program provides class and small group instruction as well as structured reading practice and has been shown to significantly improve measures of reading comprehension (Hasselbring and Glaser, 2000).

The purpose of the current study is to examine the potential impact of using a stationary bike with a desktop (FitDesk) in comparison to a traditional desk in a high school English class using the READ 180 program. We hypothesize that the students’ performance scores on the READ 180 program will not differ between the two desk types. We also examine the potential impact of using the FitDesk on subjective measures related to reading and schoolwork. Due to the paucity of literature in this area, we are unable to develop hypotheses for these measures.

## Methods

### Participants

The study took place in an urban secondary school in a large city in southeastern United States. The participants were students in a ninth-grade basic English class designed for below average readers to help the students improve their reading skills. Twenty students, 13 males and 7 females with an average age of 14.55 (*SD* = 0.67), completed the study. The participants self-identified as 70% African American, 15% White, 10% Hispanic, and 5% other. Participants were recruited by their teacher who used documentation from the researchers to explain the study to the students and parents. Consent forms were signed by both the parents and student volunteers. All participants were in good health and able to pedal a stationary bicycle continuously for 40 min. The study was approved by the university’s institutional review board.

### Procedures

This study compared the use of an active workstation (FitDesk) and a traditional school desk during a normal literature/reading class period. A FitDesk is a stationary bike with a desktop that allows students to read or do schoolwork during class while pedaling at a comfortable pace. A crossover research design was used across 16 weeks resulting in each student using a FitDesk for 8 weeks and a traditional desk for 8 weeks. Half of the students were randomly assigned to the FitDesk group for the first 8 weeks of the study and then reassigned to the traditional desk group for the second 8 weeks of the study and vice versa. The first 8-week session took place between October and December of the school year. The second 8-week session took place between January and March of the school year. This resulted in a 4 week break between the two 8-week sessions during the Christmas holidays.

The participants completed all measures (see below) at the beginning and end of each 8-week session resulting in a pre and post measure for each desk condition. All measures were completed at a traditional desk under teacher supervision.

The students followed a READ 180 program and completed their normal classroom activities in a 90-min literature/reading class for the duration of the research study. READ 180 is a learning intervention program designed for students to improve reading comprehension, vocabulary, and writing skills. The FitDesk group worked for 40 min on the FitDesks for the reading assignments and small group instruction portions of the READ 180 program and then worked for the remaining 50 min of the class period seated at standard classroom desks. The traditional desk group worked the entire 90-min class period seated at standard classroom desks.

### Measures

#### Scholastic Reading Inventory

The READ 180 program includes the Scholastic Reading Inventory (SRI), a computerized assessment of reading comprehension and proficiency. The SRI requires students to answer multiple choice and fill-in-the-blank questions after reading a short passage and is administered as part of the normal class assessment procedure multiple times during the year to evaluate student progress. Lexile scores are produced based on each student’s performance on the SRI and are used to determine each student’s current reading level. For reference, the 25th–75th percentile Lexile score range for students in the ninth grade is 1,040L–1,350L.

The SRI has been validated with students with disabilities using the Stanford Diagnostic Reading Test and with all types of students using the Stanford Achievement Tests (Stebbins et al., 2012). A correlation of 0.65 was found between the SRI and the Stanford Diagnostic Reading Test while correlations between 0.79 and 0.82 were found between the SRI and the Stanford Achievement Test (Scholastic Inc, 2007).

#### Subjective Measures

The subjective survey included a 38-item Visual Analog Scale (VAS) assessing factors related to reading and schoolwork (e.g., positiveness, commitment, motivation) as well as physiological reactions (e.g., feelings of restlessness). The VAS was administered on a computer screen and used the standard scale from 0 (not at all) to 100 (extremely). The students could slide a marker across the scale to provide a visual and numerical answer to each question. For example, for the question “How motivated to read were you?” the student could move the marker on the scale from 0 (indicating not motivated to read at all) to 100 (indicating extremely motivated to read).

### Data Analysis

All data were analyzed using the IBM SPSS statistical analysis program (SPSS 27; SPSS Inc., Chicago, IL). An exploratory factor analysis was completed to determine if the VAS items were measuring similar constructs. The factor analysis used principal components extraction with direct oblimin rotation with Kaiser Normalization. The VAS items within each factor were averaged to create one component score for each factor. Significant differences in the SRI Lexile scores, and the component scores for each factor were examined using 2 (Desk type) × 2 (Pre-post) repeated measures ANOVAs. The Wilks’ Lambda results are presented for these analyses. To ensure that the study did not negatively impact reading skills in the students, a one-way ANOVA was used to examine the four SRI Lexile scores from October to March. Because the assumption of sphericity was not met, the Greenhouse-Geisser results are reported.

## Results

As shown in Table 1, the SRI Lexile scores increased in both desk conditions during the study and from the beginning to the end of the study. The 2 × 2 repeated measures ANOVA found a significant difference in Lexile scores from pre to post [*F*(1, 19) = 6.608, *p* = 0.019, $\eta_{p}^{2}$ = 0.258]. There was no significant difference in desk type [*F*(1, 19) = 0.2, *p* = 0.659, $\eta_{p}^{2}$ = 0.01] nor was there a significant interaction effect [*F*(1, 19) = 0.375, *p* = 0.548, $\eta_{p}^{2}$ = 0.019]. In addition, there was a significant difference in Lexile scores across the four testing times (October, December, January, March), [*F*(1.54, 57) = 4.758, *p* = 0.023, $\eta_{p}^{2}$ = 0.20].

**TABLE 1:** Lexile scores from the scholastic reading inventory (SRI).

		Test period	Mean	*SD*
SRI score (Lexile) by desk condition	FitDesk	Pre-test	603.05	233.59
		Post-test	639.95	245.07
	Traditional desk	Pre-test	620.80	238.83
		Post-test	644.70	229.67
Overall SRI scores (Lexile) by month		October	592.60	242.24
		December	611.40	243.66
		January	631.25	228.70
		March	673.25	226.79

The factor analysis resulted in a 4-factor solution that explains 76.72% of the variance. The four factors include a reading factor explaining 42.14% of the variance, a physiological factor explaining 15.85% of the variance, a motivation factor explaining 11.38% of the variance, and a schoolwork factor explaining 7.35% of the variance. The reading and schoolwork factors included a range of concepts related to students’ perception of reading and schoolwork during the study (Table 2). The other items in the VAS survey did not load significantly into these four factors and did not merge into other factors.

**TABLE 2:** Factor matrix after direct oblimin rotation.

Items	Reading factor	Physio- logical factor	Motivation factor	School- work factor
How well did you pay attention while doing schoolwork?	0.851			
How positive did you feel while reading?	0.945			
How clearly were you able to think while reading?	0.976			
How relaxed did you feel while reading?	0.814			
How much did you enjoy reading?	0.879			
How committed were you to reading?	0.842			
How do you rate your reading ability?	0.697			
How much effort did it take to concentrate while reading?	0.848			
How tired did you become while reading?		0.872		
How restless did you feel while doing schoolwork?		0.755		
How restless did you feel while reading?		0.756		
How motivated to learn were you?			–0.588	
How committed were you to schoolwork?			–0.641	
How much did you understand while doing schoolwork?				–0.807
How positive did you feel while doing schoolwork?				–0.716
How do you rate your learning ability?				–0.816
How much did you focus while doing schoolwork?				–0.899
How much effort did it take to concentrate while doing schoolwork?				–0.907
How much did you enjoy doing schoolwork?				–0.783

The descriptive data for each of the four factors and each item within each factor are shown in Table 3. The VAS responses for the reading, motivation, and schoolwork factors generally increased from the pre-test to the post-test. In contrast the VAS responses for the physiological factor were more mixed showing an average of no change for the FitDesk condition and a slight decrease for the traditional desk condition from the pre-test to the post-test.

**TABLE 3:** Factors and items descriptive statistics.

Factors/VAS question	Desk	Test period	Mean	*SD*
**Reading factor**	FitDesk	Pre-test	63.10	22.85
		Post-test	72.64	18.13
	Traditional desk	Pre-test	66.69	17.94
		Post-test	72.05	17.63
How well did you pay attention while doing schoolwork?	FitDesk	Pre-test	66.90	21.07
		Post-test	73.35	18.86
	Traditional desk	Pre-test	65.70	21.15
		Post-test	71.00	20.69
How positive did you feel while reading?	FitDesk	Pre-test	63.15	29.75
		Post-test	70.95	20.01
	Traditional desk	Pre-test	67.80	22.89
		Post-test	73.10	21.26
How clearly were you able to think while reading?	FitDesk	Pre-test	64.45	24.67
		Post-test	68.95	23.12
	Traditional desk	Pre-test	66.65	18.05
		Post-test	74.00	21.76
How relaxed did you feel while reading?	FitDesk	Pre-test	60.75	25.94
		Post-test	72.65	21.73
	Traditional desk	Pre-test	63.80	24.56
		Post-test	71.20	19.68
How much did you enjoy reading?	FitDesk	Pre-test	58.75	31.05
		Post-test	77.85	19.77
	Traditional desk	Pre-test	66.55	25.22
		Post-test	70.05	21.74
How committed were you to reading?	FitDesk	Pre-test	60.45	23.73
		Post-test	69.30	23.21
	Traditional desk	Pre-test	66.85	22.72
		Post-test	68.75	19.86
How do you rate your reading ability?	FitDesk	Pre-test	64.95	22.02
		Post-test	76.00	19.79
	Traditional desk	Pre-test	68.25	15.26
		Post-test	76.20	15.59
How much effort did it take to concentrate while reading?	FitDesk	Pre-test	65.40	25.11
		Post-test	72.10	20.22
	Traditional desk	Pre-test	67.90	17.36
		Post-test	72.10	17.39
**Physiological factor**	FitDesk	Pre-test	60.52	25.58
		Post-test	60.67	23.25
	Traditional desk	Pre-test	63.72	17.01
		Post-test	59.12	21.26
How tired did you become while reading?	FitDesk	Pre-test	63.75	25.69
		Post-test	65.95	25.00
	Traditional desk	Pre-test	69.00	19.15
		Post-test	62.35	28.56
How restless did you feel while doing schoolwork?	FitDesk	Pre-test	58.25	29.40
		Post-test	59.75	24.07
	Traditional desk	Pre-test	58.55	21.52
		Post-test	58.95	21.50
How restless did you feel while reading?	FitDesk	Pre-test	59.55	30.13
		Post-test	56.30	28.38
	Traditional desk	Pre-test	63.60	22.96
		Post-test	56.05	25.34
**Motivation Factor**	FitDesk	Pre-test	66.73	18.15
		Post-test	73.20	17.53
	Traditional desk	Pre-test	64.90	18.39
		Post-test	68.75	16.82
How motivated to learn were you?	FitDesk	Pre-test	66.50	24.39
		Post-test	75.50	18.72
	Traditional desk	Pre-test	65.15	20.16
		Post-test	66.30	20.69
How committed were you to schoolwork?	FitDesk	Pre-test	66.95	16.19
		Post-test	70.90	18.98
	Traditional desk	Pre-test	64.65	20.32
		Post-test	71.20	17.91
**Schoolwork factor**	FitDesk	Pre-test	66.63	21.67
		Post-test	72.53	16.67
	Traditional desk	Pre-test	68.03	17.28
		Post-test	71.52	16.08
How much did you understand while doing schoolwork?	FitDesk	Pre-test	67.30	27.13
		Post-test	73.40	18.68
	Traditional desk	Pre-test	64.55	18.26
		Post-test	71.20	19.46
How positive did you feel while doing schoolwork?	FitDesk	Pre-test	67.25	23.67
		Post-test	74.85	18.09
	Traditional desk	Pre-test	69.20	19.21
		Post-test	69.95	16.53
How do you rate your learning ability?	FitDesk	Pre-test	68.85	24.71
		Post-test	75.40	17.34
	Traditional desk	Pre-test	69.75	18.78
		Post-test	76.95	12.31
How much did you focus while doing schoolwork?	FitDesk	Pre-test	69.70	22.54
		Post-test	71.75	19.80
	Traditional desk	Pre-test	68.75	21.76
		Post-test	71.30	22.88
How much effort did it take to concentrate while doing schoolwork?	FitDesk	Pre-test	69.80	20.64
		Post-test	67.10	19.92
	Traditional desk	Pre-test	71.15	17.91
		Post-test	73.60	16.67
How much did you enjoy doing schoolwork?	FitDesk	Pre-test	59.85	27.19
		Post-test	72.65	19.49
	Traditional desk	Pre-test	64.75	22.42
		Post-test	66.10	23.03

For the reading factor, there was a significant difference from pre to post [*F*(1, 19) = 11.625, *p* = 0.003, $\eta_{p}^{2}$ = 0.380], but there was no significant difference by desk type [*F*(1, 19) = 0.193, *p* = 0.666, $\eta_{p}^{2}$ = 0.010] nor was there a significant interaction effect [*F*(1, 19) = 1.400, *p* = 0.251, $\eta_{p}^{2}$ = 0.069]. For the physiological factor, there was no significant difference from pre to post, [*F*(1, 19) = 0.413, *p* = 0.528, $\eta_{p}^{2}$ = 0.021] or desk type [*F*(1, 19) = 0.038, *p* = 0.847, $\eta_{p}^{2}$ = 0.002] nor was there a significant interaction effect [*F*(1, 19) = 0.824, *p* = 0.375, $\eta_{p}^{2}$ = 0.042]. For the motivation factor, there was a significant difference from pre to post, [*F*(1, 19) = 8.084, *p* = 0.010, $\eta_{p}^{2}$ = 0.298], but there was no significant difference by desk type [*F*(1, 19) = 1.166, *p* = 0.294, $\eta_{p}^{2}$ = 0.058] nor was there a significant interaction effect [*F*(1, 19) = 0.173, *p* = 0.682, $\eta_{p}^{2}$ = 0.009]. For the schoolwork factor, there was a significant difference from pre to post, [*F*(1, 19) = 6.322, *p* = 0.021, $\eta_{p}^{2}$ = 0.250], but there was no significant difference by desk type [*F*(1, 19) = 0.004, *p* = 0.953, $\eta_{p}^{2}$ = 0.000] nor was there a significant interaction effect [*F*(1, 19) = 0.186, *p* = 0.671, $\eta_{p}^{2}$ = 0.010].

## Discussion

The current results indicate that allowing students to use an activity workstation, such as the FitDesk, in a high school classroom does not negatively impact reading-related performance in a class designed for students who are struggling to meet the academic standards of their grade level. These results support our hypothesis that performance on the READ 180 program would not differ between desk conditions and are consistent with previous findings (Pilcher and Baker, 2016; Fedewa et al., 2017, 2018; Pilcher et al., 2017; Magnon et al., 2018; Chim et al., 2021). This suggests that activity workstations could be used in educational settings where students are required to sit for extended periods of time to help reduce sedentary behavior in children and adolescents.

Assessing students’ perception of schoolwork is a complex undertaking. For example, engagement in schoolwork is a multidimensional construct which includes academic, affective, cognitive, and behavioral components (Fredricks et al., 2004; Appleton et al., 2006; Salmela-Aro and Upadaya, 2012). The VAS items used in the current study provide a means for students to assess a number of items, including motivation/engagement as well as their perceived accomplishments related to reading and schoolwork. Although there were no significant differences based on the type of desk used, there were improvements across the study in subjective assessments related to reading, schoolwork, and motivation as indicated by the factors derived from the VAS items. This suggests that the students in this class had a positive reaction to their educational experience in a class designed for students performing below their grade level. Many factors could have contributed to this outcome including the READ 180 method, the teacher, and the opportunity to have activity workstations in the classroom. Future research can be designed to examine the impact of different aspects of the class to better delineate what may have contributed to the positive change in VAS factors shown in this study. However, it is important that the students in the current study experienced this type of positive outcome when participating in the study suggesting that they found positive aspects to their education experience.

It is not surprising that the VAS factors did not differ based on desk condition. Student effort in schoolwork as well as their motivation to complete the required tasks are related to many aspects of their lives including gender, social economic status, and teacher standards (Brookhart, 1998) and can be particularly problematical when transitioning from middle school to high school (Niehaus et al., 2012). Students’ feelings toward schoolwork, in general, are unlikely to be altered by something as simple as pedaling on a FitDesk for 40 min during one required class period. It is possible that more voluntary use of an activity workstation may have a positive benefit on students. Future studies could be designed to assess this possibility. In this study, students volunteered to be participants; however, they did not volunteer to be in that class or to complete the assignments made by the teacher, thus limiting how much choice the students actually had.

We did not expect reading performance to significantly improve based on the desk condition. Many factors contribute to the ability to maintain task performance (Hancock and Warm, 1989). Adding 40 min of light physical activity during one class is not likely to have a profound effect on academic performance. It is important to note that there was improvement in reading skills across each testing session; however, there was a decrease in reading skills during the holiday season before returning to school in January. The teachers at the school noted that this was a normal pattern for their students. Future research can more fully examine how reading skills may change across and between academic years. It is also interesting to note that although there was no significant difference in Lexile scores between the desk conditions, the students on the FitDesk showed a slightly better improvement in reading performance across the study. Although we cannot conclude that using the FitDesk improved reading performance, it is encouraging to recognize that there was no decrement in performance while using the activity workstations. Future studies could be designed to use activity workstations in multiple classes or across longer periods of time to assess whether a more prolonged experience with the workstations could improve performance across time.

Interventions that help reduce sedentary behavior are needed in many environments in developed societies (Proper et al., 2011). This is particularly important when considering the typical school setting which requires children and teenagers to be sedentary for 5–6 h (or more) each school day. The possibility of increasing physical activity in a classroom is particularly important given that many adolescents show decreasing interest in physical activity (Lubans et al., 2010). Some studies have addressed how to implement and study the potential impact of physical activity in a classroom setting; however, more research is needed (Polo-Recuero et al., 2021). The current study is one part of this over-all effort to examine how workstations can be implemented in educational settings.

The current study has some limitations. The limitations of this study are largely a side effect of doing research in a high school classroom. One limitation is the number of participants. This was determined by the classes that were available for the study. Because we were using an actual classroom setting, we were limited to the number of students in the class. There was also no control class of reading-challenged students either with the same teacher or a different teacher who did not use the READ 180 program. The crossover design used here allowed us to test the subjects under both desk conditions; however, we could not control how the students were assigned to the class, the teacher assigned to teach the class, or the use of the READ 180 program. As noted earlier in the discussion, this limits our ability to draw conclusions about the potential impact of the teacher and the READ 180 program. Future studies are needed in high school classroom settings to more fully document the potential impact of activity workstations using control classes and different performance measures. There were also some issues common in high school students, such as one student was disruptive and removed from school during the study. Finally, we had access only to students that were assigned to the class, limiting our subject pool. Additional studies are needed with more participants and using other types of academic classrooms to expand on the current results. Although the current study cannot fully address all potential questions related to using activity workstations in classroom settings it provides necessary information to help teachers and education administrators decide on how activity workstations can be implemented in a classroom setting.

## Conclusion

The current study suggests that activity workstations can be implemented in secondary education settings without negatively impacting academic performance. The current study examined reading skills in students struggling to reach grade level performance. In these students, reading skills improved across the academic year in both desk conditions. In addition, the finding that subjective assessments related to reading, schoolwork, and motivation improved during the study is encouraging. Although we cannot draw definitive conclusions based on the current study about the cause of this change in the students’ perspective related to their classes, any positive change in terms of student perceptions is promising. Finally, it is important to note that the teacher reported that the class was easier to manage and that many of the students looked forward to using the activity workstations during their assigned period. Future studies can be designed to further evaluate how activity workstations impact the classroom dynamics that could positively impact student engagement. The present findings suggest a feasible intervention that could provide a means to increase activity in students who are expected to remain seated for most of the school day. Activity workstations in the classroom provides one mechanism for students to increase their physical activity during the school day which may have long-term positive benefits on physical fitness, health, and wellbeing in the students.

## Data Availability Statement

The raw data supporting the conclusions of this article will be made available by the authors, without undue reservation.

## Ethics Statement

The studies involving human participants were reviewed and approved by the Clemson University Institutional Review Board. Written informed consent to participate in this study was provided by the participants’ legal guardian/next of kin.

## Author Contributions

JP conceived and designed the study, involved in all aspects of the study and writing the manuscript, and completed the final drafts of the manuscript. GH assisted with designing the study and establishing the methodology. JC and ED assisted with data gathering, data management, and initial data analyses. TH and PH assisted with final data management, final data analysis, and worked on earlier drafts of the manuscript. All authors contributed to the article and approved the submitted version.

## Conflict of Interest

The authors declare that the research was conducted in the absence of any commercial or financial relationships that could be construed as a potential conflict of interest.

## Publisher’s Note

All claims expressed in this article are solely those of the authors and do not necessarily represent those of their affiliated organizations, or those of the publisher, the editors and the reviewers. Any product that may be evaluated in this article, or claim that may be made by its manufacturer, is not guaranteed or endorsed by the publisher.
